# Localization and nucleotide specificity of *Blastocystis* succinyl-CoA synthetase

**DOI:** 10.1111/j.1365-2958.2008.06228.x

**Published:** 2008-06

**Authors:** Karleigh Hamblin, Daron M Standley, Matthew B Rogers, Alexandra Stechmann, Andrew J Roger, Robin Maytum, Mark van der Giezen

**Affiliations:** 1 School of Biological and Chemical Sciences, Queen Mary, University of LondonMile End Road, London E1 4NS, UK.; 2 Institute for Protein Research, Osaka University, 3-2 YamadaokaSuita, Osaka 565-0871, Japan; 3 Japan Science and Technology Agency, Institute for Bioinformatics Research and Development (BIRD)4-1-8 Honmachi, Kawaguchi, Saitama 332–0012, Japan; 4 Department of Biochemistry and Molecular Biology, Dalhousie University5850 College St., Halifax, Nova Scotia B3H 1X5, Canada

## Abstract

The anaerobic lifestyle of the intestinal parasite *Blastocystis* raises questions about the biochemistry and function of its mitochondria-like organelles. We have characterized the *Blastocystis* succinyl-CoA synthetase (SCS), a tricarboxylic acid cycle enzyme that conserves energy by substrate-level phosphorylation. We show that SCS localizes to the enigmatic *Blastocystis* organelles, indicating that these organelles might play a similar role in energy metabolism as classic mitochondria. Although analysis of residues inside the nucleotide-binding site suggests that *Blastocystis* SCS is GTP-specific, we demonstrate that it is ATP-specific. Homology modelling, followed by flexible docking and molecular dynamics simulations, indicates that while both ATP and GTP fit into the *Blastocystis* SCS active site, GTP is destabilized by electrostatic dipole interactions with Lys 42 and Lys 110, the side-chains of which lie outside the nucleotide-binding cavity. It has been proposed that residues in direct contact with the substrate determine nucleotide specificity in SCS. However, our results indicate that, in *Blastocystis*, an electrostatic gatekeeper controls which ligands can enter the binding site.

## Introduction

*Blastocystis* is a widespread human intestinal parasite infecting up to 10% of the developed world (Stenzel and Boreham, 1996). However, the role *Blastocystis* has in causing actual disease, if any, is still a matter of dispute. It is one of the few known human parasites within the stramenopiles (Silberman *et al.*, 1996), a group also containing, for example, diatoms, oomycetes and brown algae such as the Californian giant kelp. Despite its potential pathogenic nature and its high prevalence, surprisingly little is known about *Blastocystis*. Although *Blastocystis* is a strict anaerobe (Zierdt, 1991), it has been shown to contain organelles that have features traditionally associated with mitochondria, such as cristae, a membrane potential and an associated organellar genome (Zierdt, 1991; Nasirudeen and Tan, 2004). However, these organelles also have features normally associated with anaerobic mitochondria-related organelles, such as hydrogenosomes and mitosomes (see van der Giezen *et al.*, 2005 for a review), with the apparent absence of several tricarboxylic acid (TCA) cycle enzymes and cytochromes (Zierdt, 1986).

Although classic mitochondria rely on molecular oxygen for ATP production via oxidative phosphorylation, hydrogenosomes and mitosomes occur in organisms that inhabit anaerobic environments. Anaerobic mitochondria can use a variety of substrates, for example, fumarate and nitrate, as terminal electron acceptors (reviewed in Tielens *et al.*, 2002). Other mitochondria-related organelles, referred to as mitosomes, appear to have completely lost the ability to produce energy (van der Giezen *et al.*, 2005). In contrast, hydrogenosomes use protons instead of oxygen as terminal electron acceptors, producing ATP, acetate, carbon dioxide and hydrogen from pyruvate or malate (Müller, 1993). Hydrogenosomes lack many of the metabolic pathways traditionally associated with mitochondria and lack the classic mitochondrial electron transport chain. They can, however, utilize substrate-level phosphorylation for ATP production via succinyl-CoA synthetase (SCS), an enzyme that is also found in the mitochondrial TCA cycle (Dacks *et al.*, 2006).

Succinyl-CoA synthetase is composed of two subunits, alpha and beta, which generally form dimers in eukaryotes and tetramers in prokaryotes (Bridger *et al.*, 1987). The substrate-level phosphorylation reaction catalysed by SCS is thought to take place via three steps. In the forward direction, this is first the formation of a non-covalent enzyme–succinyl-phosphate complex from succinyl-CoA and inorganic phosphate with the concomitant release of CoA (see step 1 below). The next step (2) is the formation of a covalent phosphoryl-enzyme intermediate with the release of succinate. The final step (3) uses the high transfer potential of the phosphoryl-enzyme intermediate to phosphorylate the NDP substrate, forming NTP.

(1)
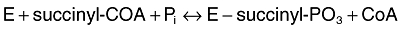


(2)
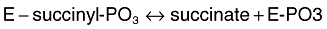


(3)
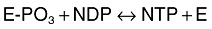


**Fig. 1:**
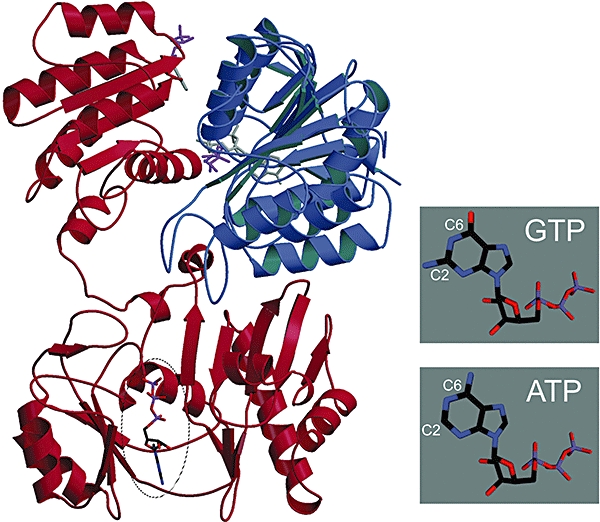
Structural model of *Blastocystis* SCS. The homology model of *Blastocystis* SCS, using pig SCS (PDB code 2fp4) as a template, is shown with the alpha subunit in blue and the beta subunit, containing the nucleotide-binding region, in crimson. The ligands GTP (surrounded by oval in bottom, front) and co-enzyme A (top, rear) are taken from the template and from *E. coli* SCS (PDB code 1jkj) respectively. The inset on the right shows GTP (top) and ATP (bottom) as stick figures, with carbons 2 and 6 on the 6-membered ring indicated. The figure was prepared using Molscript v2.1.2 (Kraulis, 1991) and Raster3 (Merritt and Bacon, 1997).

Whereas the prokaryotic homologues are capable of using both types of nucleotides, eukaryotic SCS enzymes usually occur as different GTP- and ATP-specific isoforms (Bridger *et al.*, 1987). As illustrated in Fig. 1, the nucleotide-binding site of SCS lies in the N-terminal domain of the beta subunit. The specificity for ATP or GTP is expected to result from interactions with two differing substitutions on the six-membered ring of the nucleotide. The first is at carbon 2; in GTP, this carbon is bound to an amino group but, in ATP, it is unsaturated. The second is at carbon 6; in GTP, it is bound to a carbonyl oxygen but, in ATP, it is bound to an amino group. Previous work by Fraser *et al.* implicated Gln 20 β, which is hydrogen-bonded to the carbonyl oxygen of GTP in pig SCS, as the primary residue responsible for selecting GTP over ATP (Fraser *et al.*, 2000; 2006).

Most of the research into the biochemistry and structure of eukaryotic SCS comes from animal model organisms, such as pig (Fraser *et al.*, 2000; 2002). The limited information on protistan SCS suggests that eukaryotic SCS homologues are more diverse than generally presumed. For example, the *Trichomonas vaginalis* SCS (Lahti *et al.*, 1992; 1994) appears to form a tetramer that can use both GTP and ATP, similar to its prokaryotic homologues (Jenkins *et al.*, 1991).

We have characterized the *Blastocystis* SCS because, in the absence of a classic mitochondrial electron transport chain, it is likely to be one of the main ATP producing enzymes in this parasite. Here we present the genes encoding both alpha and beta SCS subunits, their phylogenetic analysis, the cellular localization of SCS and a biochemical characterization of this enzyme. In order to investigate the question of nucleotide specificity, a structural model of *Blastocystis* SCS was built, based on pig SCS. Induced-fit docking calculations and molecular dynamics (MD) simulations of unbinding were performed using both ATP and GTP ligands. Our work suggests that residues outside the catalytic site control nucleotide specificity via an electrostatic gatekeeper mechanism.

## Results and discussion

### Identification and characterization of *Blastocystis* SCSα and SCSβ genes

The lengths of the *Blastocystis* SCS alpha and beta subunits are 318 and 416 residues respectively. The predicted molecular weights of the alpha and beta subunits are 33.4 and 45.1 kDa, respectively, with corresponding theoretical isoelectric points of 9.34 and 8.47. These predicted sizes and values are similar to SCS proteins from other organisms.

A comparison of the genomic and cDNA sequences of the SCS subunits revealed the presence of seven introns (Table S1); these are the first introns to be reported in *Blastocystis*. Two introns are present in SCSα and five in SCSβ. Interestingly, these introns are very small and surprisingly consistent in length, only 30–35 bp, placing them within the smaller-size bracket of stramenopile introns. Highly expressed genes have been shown to contain small introns (Castillo-Davis *et al.*, 2002). In *Blastocystis*, expressed sequence tags (ESTs) encoding SCS are as abundant as those encoding glycolytic enzymes (see Table S2). This indicates that SCS is highly expressed and likely plays an important role in the overall metabolism of this parasite.

### *Blastocystis* SCS is localized to the mitochondria-like organelles

A Clustal W alignment (Chenna *et al.*, 2003) comparing *Blastocystis* SCS with its prokaryotic and eukaryotic homologues highlighted the presence of putative N-terminal targeting signals (Fig. S1). These are comprised of positive and hydrophobic residues with very few negative residues as seen in typical hydrogenosomal and mitochondrial targeting sequences. The *Blastocystis* SCS leader sequences also have arginine at position −2 from the putative cleavage site which is very common in mitochondrial and hydrogenosomal targeting signals (van der Giezen *et al.*, 2005). The subcellular localization prediction tools Mitoprot (Claros and Vincens, 1996) and TargetP 1.1 (Emanuelsson *et al.*, 2000) both predict a mitochondrial localization for both subunits.

**Fig. 2:**
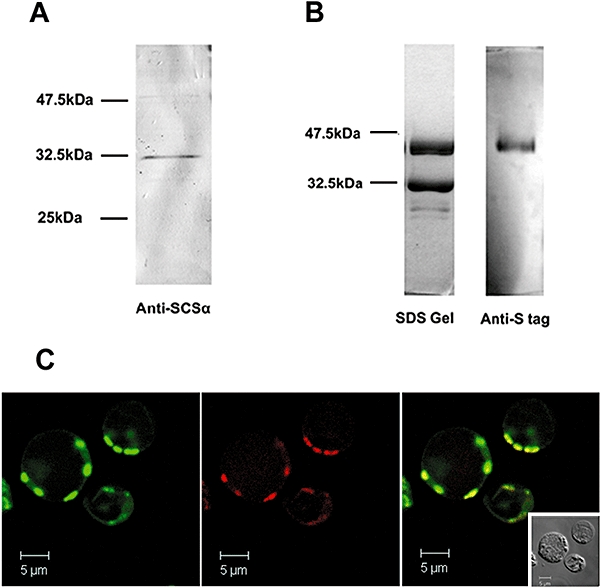
Localization of *Blastocystis* SCS. A. Western blot of *Blastocystis* whole-cell lysate (13.6 μg) probed with antiserum raised against pig SCSα indicating the specificity of the antiserum. A strong cross-reacting band of approximately 32.5 kDa is recognized, in agreement with the predicted molecular weight of 31.5 kDa. B. Coomassie-stained SDS polyacrylamide gel (left) showing the results of His-tag purification of *Blastocystis* SCS. Two major bands are present; a lower band of a size consistent with the His-tagged SCSα and a higher band with a size consistent with a S-tagged SCSβ. Probing of this sample with an S-protein antibody (right) confirmed that the higher band was indeed SCSβ, indicating that the two *Blastocystis* SCS subunits do interact. C. Intracellular distribution of *Blastocystis* SCS using confocal microscopy. Cells were double-labelled for SCS (left) and the mitochondrial marker MitoTracker (middle). SCS clearly colocalizes with MitoTracker (right).

An anti-pig-SCSα antibody was used to test the putative mitochondrial localization of *Blastocystis* SCS. This antibody produced a strong cross-reacting band of approximately 32 kDa on a Western blot of *Blastocystis* whole-cell lysate (Fig. 2A). Confocal microscopy showed the pig anti-SCSα colocalized with Mitotracker orange, a mitochondrion-specific stain (Fig. 2C).

Unfortunately, neither the anti-pig-SCSβ antibody nor the anti-*Neocallimastix*-SCSβ antibody recognized *Blastocystis* SCSβ. However, given the presence of a typical targeting signal and the presence of the alpha subunit in the mitochondria, the beta subunit is likely to be localized to the mitochondria as well. Both subunits were expressed together in tagged forms using a 6xHis tag for SCSα and an S tag for SCSβ. Affinity purification of the expressed proteins showed the co-purification of the α–β complex using either a nickel-sepharose column specific for the His tag (as shown in Fig. 2B) or an S tag-specific column (results not shown). These results clearly indicate that the two *Blastocystis* subunits interact as expected.

Eukaryotic SCSs are typically found in mitochondria and the localization of the *Blastocystis* SCS is therefore not unexpected. However, in this case, it provides insight into the metabolism of the enigmatic mitochondria-like organelles in this parasite. Its presence suggests that the organelle is indeed involved in energy metabolism and therefore unlikely to be a mitosome as per definition (see Dacks *et al.*, 2006). Our recent analysis of over 12 000 ESTs (Stechmann *et al.*, 2008) suggests that *Blastocystis* does not use a normal TCA cycle. In agreement with biochemical data (Zierdt *et al.*, 1988), our EST analyses indicate that only the latter half of the TCA cycle is present. This combination of enzymes is known as the malate dismutation pathway (van Hellemond *et al.*, 2003). This pathway runs in the opposite direction compared with the TCA cycle and uses fumarate as an electron sink. The end-product of this pathway is succinate and SCS is not part of this route. However, organisms containing a malate dismutation pathway normally have an enzyme called acetate : succinate-CoA transferase (ASCT) which forms an energy-conserving shuttle with SCS (van Hellemond *et al.*, 2003). We have identified a *Blastocystis* EST for ASCT in agreement with this observation. This enzyme is found in mitochondria and hydrogenosomes of only a few organisms (van Hellemond *et al.*, 1998; van Grinsven *et al.*, 2008). ASCT allows acetyl-CoA to be used as a CoA donor to form succinyl-CoA from succinate. SCS then conserves the high-energy potential of the succinyl-CoA thioester bond by forming ATP from ADP and P_i_.

### Phylogenetic analysis of SCS subunits

In order to investigate whether *Blastocystis* SCS is ATP- or GTP-specific, we conducted phylogenetic analyses to see if it falls into one of the families that have been well characterized. SCS alpha and beta subunits are classic mitochondrial marker proteins and have previously been characterized from both mitochondrion- and hydrogenosome-containing eukaryotes (Lambeth *et al.*, 2004; Dacks *et al.*, 2006).

The SCS alpha and beta subunit phylogenies group the *Blastocystis* sequences among other eukaryotic SCSs, but fail to resolve the position of *Blastocystis* within the eukaryotes (Figs. S2 and S3). This inability to clearly resolve the position of the *Blastocystis* SCS subunits may result from a lack of a more comprehensive sampling of stramenopile and chromistan sequences. In both trees, alpha-proteobacteria are sister to eukaryotes, indicating the mitochondrial ancestry of this protein. Several studies have suggested that *Rickettsia prowazekii* is the sister taxa to mitochondria (see for example Karlin and Brocchieri, 2000). Interestingly, *R. prowazekii* is not the immediate sister to the eukaryotes in our analyses, in line with more comprehensive studies regarding the relationship between alpha-proteobacteria and mitochondria (Wu *et al.*, 2004).

As previously described by Lambeth *et al.* (2004), there are two clearly distinct clades of animal SCSβ corresponding to the different affinity for GTP and ATP exhibited by animal SCS. In addition to the cases discussed in the Lambeth *et al.* (2004) study, we find that several groups of unicellular eukaryotes also encode distinct forms of SCSβ. *Dictyostelium discoideum*, *Naegleria gruberi*, *Tetrahymena thermophila* and an *Aspergillus* all have two SCSβ isoforms. Other well-sampled groups, such as plants, show evidence for only one isoform of SCSβ. Taken together, it is clear that a taxonomically broad range of eukaryotes possess two distinct forms of SCSβ, suggesting that nucleotide specificity might be deduced by phylogenetic analyses.

However, only one type of SCSβ was found among the *Blastocystis* ESTs. The completed genomes of other stramenopiles were queried and only one type of SCSβ was found in each of these genomes as well. Therefore, it is unfortunately impossible to tell from our phylogenetic analyses whether the *Blastocystis* SCS is ATP- or GTP-specific.

### *Blastocystis* SCS is ATP-specific

**Fig. 3:**
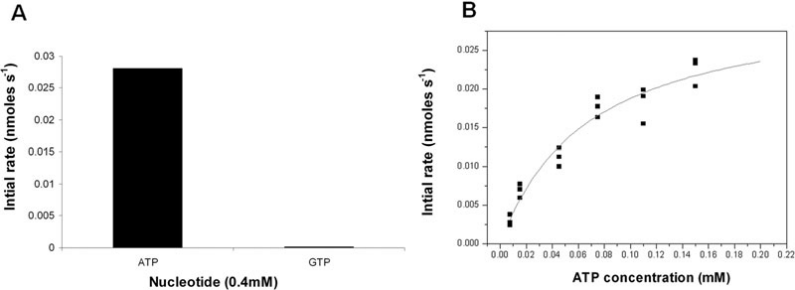
*Blastocystis* SCS enzyme kinetics measured in the direction of succinyl-CoA formation. A. Comparison of the initial rate of SCS with ATP and GTP under standard assay conditions (Fraser *et al.*, 2002). No detectable rate was seen for GTP. B. Michaelis–Menten plot for *Blastocystis* SCS in the presence of ATP. Graph shows the initial rate (nmol s^−1^) versus ATP concentration (mM). The *Blastocystis* SCS K_m_ and V_max_ were determined by curve fitting to the hyperbolic M-M equation, giving values of 68 μM and 32 nmol s^−1^ respectively.

Primary sequence analysis of the *Blastocystis* SCS suggested that the enzyme is GTP-specific because of the presence of glutamine at the equivalent position to Gln 20 β in pig GTP-specific SCS (Fraser *et al.*, 2006). The initial rates of reaction for SCS with GTP and ATP were compared under standard reaction conditions, as defined by Fraser *et al.* (2002), using 0.4 mM of each nucleotide. Under these conditions, the recombinant *Blastocystis* SCS showed no detectable activity with GTP, but a rate of 0.028 nmol s^−1^ with ATP, as shown in Fig. 3A. The enzyme therefore has specificity for ATP rather than GTP, in contrast to our initial sequence analysis predictions based on the proposals of Fraser *et al.* (2006).

The ATP activity was then further characterized, as shown in Fig. 3B. The *Blastocystis* SCS has a K_M_ of 68 μM for ATP, which is comparable to ATP-utilizing SCS enzymes from other species such as *Escherichia coli*, which has an ATP K_m_ of 70 μM (Fraser *et al.*, 2002). However, the *Blastocystis* SCS K_cat_ is only 133 min^−1^ in comparison with the *E. coli* SCS K_cat_ of 2684 min^−1^ (Fraser *et al.*, 2002). A similar decrease in catalytic rate, with no effect on substrate binding (K_m_) was observed when recombinant pig SCS was compared with the native enzyme (Fraser *et al.*, 2000). The substrate specificity shown in our enzyme assays suggests that *Blastocystis* SCS is more similar to its mammalian ATP-specific homologues than the protistan *T. vaginalis* SCS (Steinbüchel and Müller, 1986) which can use both ATP and GTP (Jenkins *et al.*, 1991).

The SCS sequences previously examined (human, mouse, pig, pigeon, *E. coli* and *Caenorhabditis elegans*) appear to support the role of Gln 20 β in determining nucleotide selectivity, as Gln is present in all of the GTP-specific enzymes, while the ATP-specific and non-specific forms have Pro at this position. However, the appearance of Gln at position 20 in *Blastocystis* SCS coupled with its ATP specificity indicates that it is not this residue that dictates nucleotide specificity.

Considering the problem of nucleotide specificity in general, Nobeli *et al.* (2001) proposed that the distribution of hydrogen bond donors and acceptors could be used to distinguish ATP-specific binding sites from GTP-specific binding sites. There are 17 residues that have close contacts with GTP in pig SCS, of which all but three are conserved in *Blastocystis* SCS; Leu 109 β in pig SCS corresponds to Val, Asp 110 β corresponds to Lys and Phe 219 β corresponds to Leu in *Blastocystis* SCS. Moreover, as all three of these residues make only backbone contacts to GTP, it is not obvious how these substitutions would alter the specificity of the enzyme for a particular nucleotide, especially considering the fact that the SCS binding site is very flexible (Fraser *et al.*, 2000; 2006).

### Molecular modelling of nucleotide specificity

**Fig. 4:**
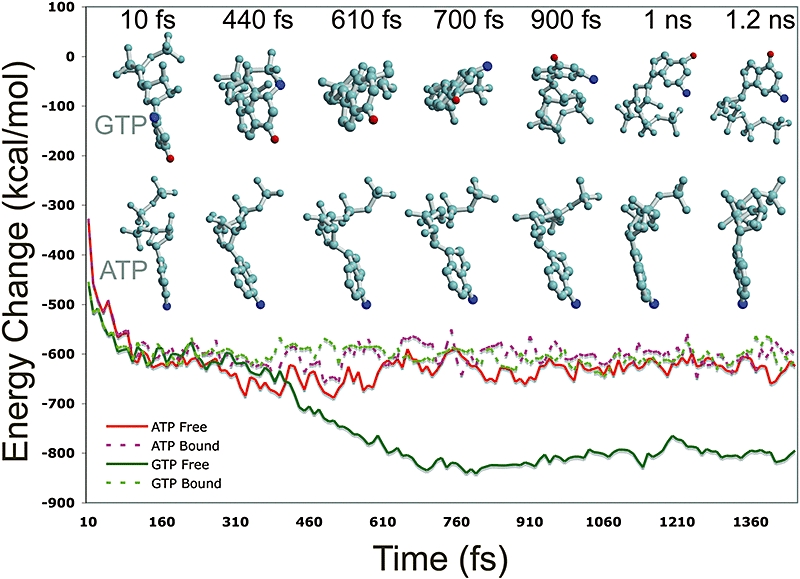
Early stages of unbinding for ATP and GTP from *Blastocystis* SCS. Starting from the bound state, the first 1.5 ns of two MD simulations (Free and Bound) are shown for each nucleotide. In the Free simulation, the ligand was allowed to diffuse away from the receptor. In the Bound simulation, the ligand was restrained to its initial position by a harmonic penalty function, as described in *Experimental procedures*. The energy change was measured relative to the ligand-free receptor. Seven snapshots of the ligand trajectories were taken in the first 1.5 ns, illustrating the flipping of GTP. These snapshots were taken from the same viewpoint, with the receptor cavity located below the ligand, and were translated in order to fit into the figure. The figures were prepared using Molscript v2.1.2 (Kraulis, 1991) and Raster3 (Merritt and Bacon, 1997).

In order to investigate the origins of nucleotide specificity in *Blastocystis* SCS, we built a homology model based on GTP-specific pig SCS (Fig. 1). We then constructed hypothetical models of the ATP-*Blastocystis* SCS and GTP-*Blastocystis* SCS complexes using flexible docking. The docking results indicated a modest preference for ATP, but did not rule out GTP binding (Fig. S4). In order to observe the protein–ligand interactions more realistically, we carried out unbinding simulations for ATP and GTP starting from the predicted complex models. The unbinding trajectories of the two nucleotides were very different (see Fig. 4). Upon unbinding, the six-membered ring in GTP flipped 180 degrees so that the carbonyl group was pointing away from the binding site. This change was accompanied by a significant drop in the energy of the GTP-*Blastocystis* SCS system, as indicated in Fig. 4. ATP, on the other hand, diffused away from the binding site without altering its nucleotide orientation and without a significant drop in energy. In order to validate these results further, the induced-fit docking and unbinding procedures were repeated using the pig SCS nucleotide-binding site. As Fig. S4 shows, the docking score strongly favours GTP in this case, and the unbinding simulations (Fig. S5) did not indicate a significant conformational change or associated drop in energy upon unbinding.

**Fig. 5:**
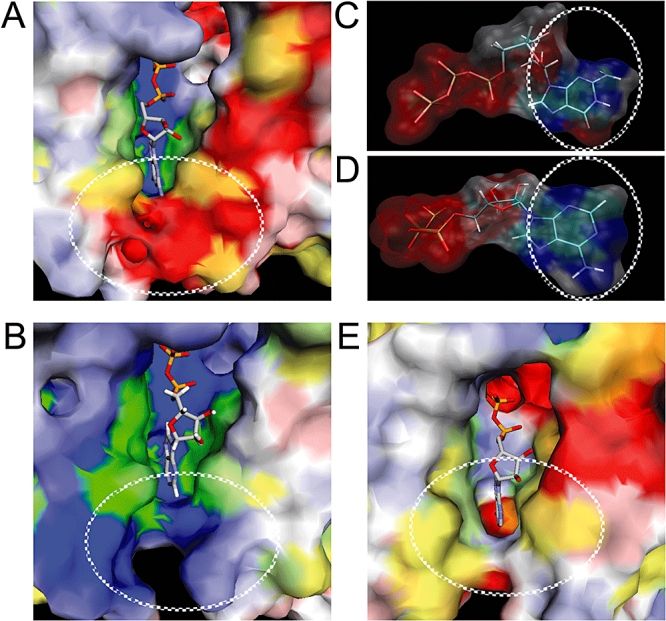
Electrostatic surfaces of nucleotide-binding region. The electrostatic surfaces of pig SCS (A) and *Blastocystis* SCS (B) are shown with the charged rim indicated by white ovals. Semi-transparent electrostatic surfaces of GTP (C) and ATP (D) are shown with the differing regions, where GTP possesses a dipole that is approximately orthogonal to the main dipole moment, indicated by a white dotted oval. For reference, the electrostatic surface of *E. coli* SCS is shown as well (E), with the neutral rim indicated by a white oval. The electrostatic surfaces were prepared using the eF-surf server (Kinoshita, 2006, http://ef-site.hgc.jp/eF-surf/) and eF-site (Kinoshita and Nakamura, 2004).

The change in energy of the GTP-*Blastocystis* SCS system upon unbinding was due almost entirely to the electrostatic part of the solvation energy. Basu *et al.* (2004) has argued that electrostatic forces are sufficient for discriminating between ATP and GTP binding. Our detailed modelling studies of *Blastocystis* SCS support this argument. Both ATP and GTP possess a strong dipole moment pointing from the phosphate groups to the nucleotide region. In GTP, however, there is a second dipole, approximately orthogonal to the first, pointing from the carbonyl oxygen at carbon 6, to the amino group at carbon 2 (see Fig. 5). Unlike hydrogen bonds, which act over a short distance range, electrostatic forces are long-range, and extend into the solvent layer surrounding the binding site. Therefore, residues that are not necessarily in direct contact with the ligand could have profound effects on ligand stability. In order to understand the origin of the charges, electrostatic surfaces of the *Blastocystis* SCS, pig SCS and *E. coli* SCS binding sites were constructed. As Fig. 5 shows, the electrostatic surface surrounding the nucleotide-binding region is positive in *Blastocystis* SCS, whereas the corresponding region in the pig GTP-specific SCS is negative. These differences are due to two changes that do not involve direct side-chain contacts with the nucleotide, Glu 42 β to Lys and Asp 110 β to Lys. Interestingly, in the non-specific *E. coli* SCS, the corresponding residues are Pro and Asp and the surface is neutral and hydrophobic (see Fig. 5), consistent with a scenario where both ATP and GTP can bind but GTP is preferred. The rim surrounding the binding site in *Blastocystis* SCS thus acts as an electrostatic gatekeeper that prevents GTP from orienting in the bound conformation.

The paradigm for changing co-enzyme specificity is by engineering discriminating interactions within the binding pocket, as shown by studies changing NAD^+^ to NADP^+^ specificity in dehydrogenases (Scrutton *et al.*, 1990; Chen *et al.*, 1996; Lunzer *et al.*, 2005). Interestingly, however, an empirical study of ATP/GTP discrimination in known structures suggested ‘fuzzy clustering’ of interactions in the nucleotide-binding sites with no clear common discriminatory motif for the two nucleotides (Nobeli *et al.*, 2001). In *Blastocystis*, SCS appears to have retained a binding pocket capable of binding either substrate and evolved an alternate specificity mechanism by changing key residues controlling access to the active site.

The flexible docking simulations provide another important clue regarding nucleotide specificity. As observed by Fraser *et al.* in the GTP-specific pig SCS structure, Gln 20 β is in such a conformation as to stabilize the buried GTP carbonyl group and would thus interfere with the amino group in ATP (Fraser *et al.*, 2000; 2006). In the initial homology model of SCS, the side-chain of Gln 20 β does indeed interfere with ATP binding. However, in the induced-fit model, the side-chain has moved out of the way, allowing ATP to dock deep within the binding site, similar to the *E. coli* SCS binding mode. This movement is, in turn, possible because a space has been opened by replacement of large hydrophobic groups (Phe 219 β and Leu 109 β) with relatively smaller ones: Leu and Val respectively. These differences are indicated in Fig. S6.

### Concluding remarks

Although SCS was first purified in 1955 and has been studied in depth for more than 50 years (Kaufman and Alivisatos, 1955), studies in diverse organisms are providing additional insights into this TCA cycle enzyme. Here we have broadened the taxonomic breadth of SCS research and characterized the first stramenopile SCS from the human intestinal parasite *Blastocystis*. The mitochondria-like organelles from this anaerobe are devoid of cytochromes (Zierdt, 1986) and SCS seems to play an important role in its energy metabolism. Further studies are needed to unravel the biochemistry of SCS, the only enzyme in the TCA cycle that directly produces ATP. Recent experimental evidence supports a hypothesis where ATP specificity is linked with energy conservation, while GTP specificity is linked with the reverse reaction in order to produce succinyl-CoA (Lambeth *et al.*, 2004).

Although previous work in higher organisms suggested that the presence of Glu 20 in the active site was necessary for GTP activity, we find here that it is also compatible with ATP activity. Furthermore, our finding that GTP is destabilized by interaction with Lys 42 and Lys 110 suggests that long-range electrostatic interactions that penetrate into the solvent layer, here referred to as an ‘electrostatic gatekeeper’, can influence nucleotide selectivity. These findings, along with the recent work by Basu *et al.* (2004), raise the question of whether such a mechanism might be general for nucleotide specificity in other enzymes and in other organisms. If so, this work indicates that residues that are not in direct contact with a substrate can play an important role in substrate specificity.

## Experimental procedures

### Organisms and culture conditions

DNA and cDNA from *Blastocystis* strain NandII, obtained from the American Type Culture Collection (ATCC 50177), was used in this study. Human *Blastocystis* sp. isolate DMP/02-328 was obtained during routine screening and was grown at 36°C with a mixed bacterial flora in LYSGM with 5% adult bovine serum. LYSGM is a modification of TYSGM-9 in which the trypticase and yeast extract of the latter are replaced with 0.25% yeast extract (Sigma) and 0.05% neutralized liver digest (Oxoid). Subtyping of *Blastocystis* sp. DMP/02–328 indicated that this strain is subtype 4 (Stensvold *et al.*, 2007) whereas *Blastocystis* sp. NandII is subtype 1.

*Escherichia coli* strain α select silver efficiency (Bioline) was used for cloning and BL21(DE3) pLysS (Bioline) was used for protein expression. Both strains were grown in Luria–Bertani broth at 37°C.

### Identification of SCSα and SCSβ genes

Putative SCSα and SCSβ genes were identified in the *Blastocystis hominis* EST project (http://amoebidia.bcm.umontreal.ca/pepdb) using blastn with the ESTs as queries. Full-length genes were obtained by 5′ and 3′ rapid amplification of cDNA ends using the GeneRacer kit (Invitrogen). SCSα was amplified from genomic DNA using the forward and reverse primers, 5′-ATG CTT TCC CGT GTA TCG CAG G-3′ and 5′-TTA AGC CTT TCC AGC AGC C-3′. SCSβ amplification from genomic DNA was carried out with the forward primer, 5′-ATG CTG AGA ATG GCC CCT AAG AC-3′ and the reverse primer 5′-GGC TGT TGC TTC TCT GAA GCA CTA A-3′; both amplifications used Immomix polymerase (Bioline). Polymerase chain reaction (PCR) products were purified using QuickClean (Bioline) and TA cloned into pGEM-T-Easy (Promega). *Blastocystis* SCS sequences are deposited into GenBank with the following accession numbers: alpha subunit cDNA, EU076378; alpha subunit genomic sequence, EU090061; beta subunit cDNA, EU076379; beta subunit genomic sequence, EU076380. Clustal W alignments (Chenna *et al.*, 2003) and subcellular localization servers Mitoprot (Claros and Vincens, 1996) and TargetP 1.1 (Emanuelsson *et al.*, 2000) were used to predict mitochondrial targeting signals.

### Phylogenetic methods

Multiple sequence alignments for both SCSα and SCSβ were generated from a representative selection of eukaryotic taxa. New sequences were added to datasets previously reported in Dacks *et al.* (2006). Sequences were retrieved from public databases, including GenBank, JGI and TIGR (accession numbers available upon request). Multiple sequence alignments were constructed using Clustal X (Thompson *et al.*, 1997) and then manually adjusted using MacClade 4.06. SCSα and SCSβ trees were reconstructed from 265 and 313 unambiguously aligned amino acid positions respectively. Preliminary alignments were analysed using Tree-puzzle 5.2 (Schmidt *et al.*, 2002) and taxa that failed amino acid composition tests were removed from further analysis. Bayesian phylogenies were generated using Mr Bayes 3.1.2 (Huelsenbeck and Ronquist, 2001; Ronquist and Huelsenbeck, 2003) from 1 000 000 generations divided between two parallel runs of 500 000 generations, each with sampling every 100 generations. Although the likelihoods for both subunit trees reached a plateau rapidly, 100 burnin trees were nevertheless removed in computing the posterior probabilities for branches. The substitution model was inferred using a mixed model of amino acid substitution and rate across site variation was modelled using a discrete gamma distribution with four gamma categories and one category of invariable sites. Bootstraps were generated using PHYML 2.4.5 from 100 replicates (Guindon *et al.*, 2005) using the WAG substitution model and rates across sites variation modelled as described above.

### Succinyl-CoA synthetase expression and purification

Succinyl-CoA synthetase genes were amplified from cDNA without their targeting sequences for protein expression. The SCSα open reading frame (ORF) was amplified using the forward primer 5′-aga aga *GGA TCC* GAG CAG CAC TGC CCG TGT GTG GG-3′ and the reverse primer 5′-tct tct *GCG GCC GC* T TAA GCC TTT CCA GCA GCC TTC-3′ which added *Bam* HI and *Not* I restriction sites (indicated in italics) respectively. The SCSβ ORF was amplified using the forward primer 5′-aga aga *CAT ATG* CTG AGA ATG GCC CCT AAG ACT GTG-3′ and reverse primer 5′-tct tct *GTC GAC* GTG CTT CAG AGA AGC AAC AGC C-3′ which added *Nde* I and *Sal* I restriction sites respectively.

Amplification from cDNA was carried out with Phusion polymerase (NEB) yielding amplicons of the expected size, roughly 0.9 kb for SCSα and 1.2 kb for SCSβ. PCR products were purified using Qiagen Gel Extraction kit and subsequently digested with their respective restriction digestion enzymes and cloned sequentially into pCDF duet (Novagen). The pCDF duet vector added a 6XHis tag to the N-terminus of SCSα and a S tag to the C-terminus of SCSβ. The SCSαSCSβ pCDFduet plasmid was purified using Qiagen miniprep kits, sequenced to confirm its validity (MWG) and used to transform *E. coli* BL21(DE3) pLysS (Bioline) cells.

Cells were grown to exponential phase and protein expression was induced overnight at 16°C with 0.4 mM IPTG. Cells were pelleted by centrifugation at 3500 *g* for 20 min at 4°C and resuspended in lysis buffer (20 mM sodium phosphate buffer pH 7.4, 0.5 M NaCl, 20 mM imidazole). Cells were broken with a Sonics Vibracell Ultrasonic processor. Cell debris was removed with centrifugation at 35 000 *g* for 25 min at 4°C. The supernatant was applied to a charged nickel-chelating sepharose column (Bioline) which was washed with five column volumes of lysis buffer and five column volumes of wash buffer (20 mM sodium phosphate buffer pH 7.4, 0.5 M NaCl, 50 mM imidazole). His-tagged protein was eluted with 20 mM sodium phosphate buffer pH 7.4, 0.5 M NaCl, 500 mM imidazole. The SCS complex was further purified by size exclusion using an Akta FPLC chromatography system. A pre-packed Sephacryl S-300 column (Amersham) was equilibrated with 1.5 column volumes of 20 mM sodium phosphate buffer pH 7.4. SCS was eluted in this buffer at 1.5 ml min^−1^ into 1 ml fractions. S-tag protein purification was performed using an STag Purification kit (Novagen).

### Western blotting

*Blastocystis* whole-cell protein lysate, from strain DMP/02-328, was separated on a 10% SDS polyacrylamide gel and blotted on to nitrocellulose membrane (Bio-Rad). Anti-pig SCSα (1:1000) was used as primary antibody followed by anti-rabbit HRP conjugate (Pierce) 1:10 000 as a secondary antibody. S-protein HRP conjugate (Novagen) was used to detect overexpressed SCSβ at a 1:5000 dilution. Signal was detected using CN/DAB Substrate Kit (Pierce).

### Immunolocalization of SCSα

*Blastocystis* cells and strain DMP/02-328 were stained with 500 nM MitoTracker orange (Invitrogen) for 30 min and washed in 1× PBS. Cells were fixed with formaldehyde at 37°C for 15 min and permeabilized with ice-cold acetone for 5 min. After washing in PBS with Tween-20, cells were blocked with 1% BSA in PBS for 1 h at room temperature. Slides were incubated with anti-pig SCSα antibody (1:100 dilution) for 1 h at 37°C, washed thoroughly in PBS with Tween-20 and incubated in secondary anti-rabbit antibody conjugated to AlexaFluor 633 (Invitrogen) for 1 h at 37°C (1:200 dilution). Images were viewed using a Zeiss LSM 510 meta laser-scanning confocal microscope with a 63x objective (oil immersion) and a 1 Airy unit confocal pinhole. Images were captured using LSM510 PCM software.

### Enzyme assays

The SCS enzyme assays were carried out as described by Bridger *et al.* (1969) and Fraser *et al.* (2002) with minor modifications. Briefly, the enzyme assay solution contained 129 μM CoA, 10 mM succinate, 50 mM KCl, 10 mM MgCl_2_ and 50 mM Tris HCl pH 7.4. Standard reaction conditions also included 0.4 mM ATP or GTP. For determination of the SCS kinetic parameters for ATP, the ATP concentration was varied. The concentration of SCS protein was determined spectrometrically at A280 [extinction coefficient Abs 0.1% (= 1 g l^−1^) = 0.584] with 2.8 μM *Blastocystis* SCS added to each assay. The reaction was followed by formation of succinyl-CoA bond at 232 nm. Initial rates of reaction were measured in triplicate at 30°C.

### Structural modelling procedure

A homology model of *Blastocystis* SCS was built using the closest homologue in the Protein Data Bank (PDB), the GTP-bound form of pig SCS (PDB code 2fp4, chains A and B), as a template. Following alignment by Clustal W, the sequence identities of the alpha and beta subunits were 70% and 49%, with two and eight non-terminal gaps respectively. The alignments were then adjusted by hand to remove gaps from secondary structure segments. Side-chains that differed between *Blastocystis* SCS and pig SCS were iteratively substituted by searching for conformations from known folds deposited in the PDB that minimized clashes with other atoms. The residues at the interface of the alpha and beta subunits were more highly conserved than average (80% and 59% respectively), validating the structure of the heterodimer.

### Induced-fit docking procedure

To build an initial model of ATP-*Blastocystis* SCS, the ATP molecule was constructed from ADP in the ADP-bound form of *E. coli* SCS (PDB ID 1cqi). Partial charges for ATP were computed using Gaussian 03 (Frisch *et al.*, 2004) in vacuum without geometry optimization. The initial model was refined by a 500 fs simulated annealing MD run where the temperature was lowered linearly from 300 to 50 K, and the time step was 0.5 fs. All MD calculations were performed using the cosgene program in the myPresto simulation package (Fukunishi *et al.*, 2003; Kim *et al.*, 2003; 2004) with AMBER force field (Cornell *et al.*, 1995). The ATP molecule was fixed and the side-chains in contact with it (Q20, V44, K46, G52, G53, R54, G55, K56, V67, L69, A108, V109, K110, I111, L219, D220, K222, using 2fp4 numbering) were free to move. As 2fp4-GTP was co-crystallized with potassium, we also placed a potassium ion in the binding site in the same position as in 2fp4, although it tended to diffuse away rapidly. The simulated annealing was repeated 10 times, starting with different initial velocities, with snapshots taken every 50 fs, for a total of 90 trial complexes.

The ligand and potassium were removed from each trial complex, and the corresponding receptors were used in both rigid and flexible ligand docking calculations. All docking was performed using the sievgene program in the myPresto simulation package (Fukunishi *et al.*, 2005). The top 10 poses were retained from each docking run for a total of 1800 docked conformations. The entire procedure was repeated using GTP as the ligand, starting from the 2fp4-bound position. From the 1800 docked complexes for each ligand, one ATP complex and one GTP complex were selected for unbinding simulations (see Fig. S4). The pose with the lowest docking energy and root mean square deviation less than 3.5 Å from the initial pose was selected. Finally, the entire procedure was repeated using pig SCS (PDB code 2fp4) as the receptor.

### Unbinding procedure

Two 5 ns MD simulations (Bound and Free) were performed starting from the selected docked complexes, with a 0.5 fs time step and snapshots taken every 10 fs. In the Bound simulation, positional restraints were applied to the ligand, and the side-chains of the contacting residues (see above) were allowed to move. In the Free simulation, no positional restraint was applied to the ligand. The restraint term was then subtracted from the total energy. Both Bound and Free simulations were run using three different solvent models: GBSA, GB and vacuum.

## Supplementary material

This material is available as part of the online article from: http://www.blackwell-synergy.com/doi/abs/10.1111/j.1365-2958.2008.06228.x (This link will take you to the article abstract). Please note: Blackwell Publishing is not responsible for the content or functionality of any supplementary materials supplied by the authors. Any queries (other than missing material) should be directed to the corresponding author for the article.
