# Supplementary material for: Localization and nucleotide specificity of Blastocystis succinyl-CoA synthetase
Source: Mol Microbiol. 2008 Jun;68(6):1395–405. doi: 10.1111/j.1365-2958.2008.06228.x (PMC2440562; doi:10.1111/j.1365-2958.2008.06228.x)
Supplement: Supplementary file 1 [file mmi0068-1395-SD1.pdf]

Supplementary information

Localisation and nucleotide specificity of *Blastocystis* succinyl-CoA synthetase

Karleigh Hamblin[1§], Daron M. Standley[2,3], Matthew B. Rogers[1§], Alexandra Stechmann[4], Andrew J. Roger[4], Robin Maytum[1] and Mark van der Giezen[1§*].

[1] School of Biological and Chemical Sciences, Queen Mary, University of London, Mile End Road, London E1 4NS, United Kingdom.
[2]Institute for Protein Research, Osaka University, 3-2 Yamadaoka, Suita, Osaka 565-0871, Japan.
[3]Japan Science and Technology Agency, Institute for Bioinformatics Research and Development (BIRD), 4-1-8 Honmachi, Kawaguchi, Saitama 332-0012, Japan.
[4] Department of Biochemistry and Molecular Biology, Dalhousie University, 5850 College St., Halifax, Nova Scotia B3H 1X5, Canada.
[§] Current address: Centre for Eukaryotic Evolutionary Microbiology, School of Biosciences, University of Exeter, Stocker Road, Exeter EX4 4QD, UK.

*corresponding author
tel.: +44 1392 263 483/fax: +44 1392 263 434
e-mail: m.vandergiezen@exeter.ac.uk

Table S1 List of intron sequences present in *Blastocystis* succinyl-CoA synthetase subunits. Intron boundaries are in capitals with introns in lowercase.

Intron	sequence	start position in genomic DNA (bp)	size (bp)
SCSα 1	GAACgtaggatggcgggatcgcgtgcattaattgcttagGGTA	124	35
SCSα 2	GGAGgtggggtgttttggagcgggataagcgcgtagGCGA	276	32
SCSβ 1	AGAAgtgggtccagagcgcctggcgtcacatttagGTAC	84	31
SCSβ 2	CAGTgtagggggtcggggcgaggtgctgacgattctagTCCC	198	34
SCSβ 3	AGACgttcgcattgcgtgggtggtttcatgtgtagCCCC	335	31
SCSβ 4	CCACgtagtgctgttgccttctcttaatagctagTTTC	549	30
SCSβ 5	CAAGgtgatcgccattgcgcacattgacgcctagAGCC	919	30

Table S2 Abundance of ESTs encoding succinyl-CoA synthetase or glycolytic enzymes among 13,531 *Blastocystis* EST analysed (Stechmann et al., 2008).

Coding for	Occurrence
SCS alpha	28
SCS beta	17
hexokinase	3
Glucose-6-phosphate isomerase	7
6-phosphofructokinase	17
Aldolase	64
triose-phosphate isomerase	22
GAPDH	24
phosphoglycerate kinase	7
phosphoglycerate mutase	1
Enolase	9
Pyruvate kinase	0

Supplementary Figure legends.

Fig. S1. Succinyl-CoA synthetase targeting signals. An alignment of N-terminal amino acids of SCS alpha (A) and beta (B) subunits from *Blastocystis*, *Neocallimastix patriciarum*, *Sus scrofa*, *Escherichia coli* and *Rickettsia prowazekii* is shown. Putative or confirmed targeting signals are boxed. Amino acids were shaded according to similarity/identity scores: black indicates fully conserved residues while light grey indicates similar residues according to BOXSHADE.

Fig. S2. Phylogenetic analysis of alpha succinyl-CoA synthetase. A Bayesian tree of SCSα is shown. PHYML bootstraps are indicated above nodes, Bayesian posterior probabilities are shown below nodes. Support values for nodes with posterior probabilities below 0.5 are not shown.

Fig. S3. Phylogenetic analysis of beta succinyl-CoA synthetase. A Bayesian tree of SCSβ is shown. PHYML bootstraps are shown above nodes, Bayesian posterior probabilities are shown below nodes. Support values for nodes with posterior probabilities below 0.5 are not shown.

Fig. S4. Induced fit docking calculations. The figures show the results of 1,800 docked poses for GTP (blue) and ATP (black) using the *Blastocystis* SCS model (A) and pig SCS structure (B). Red circles indicated the best candidates, for each receptor-ligand combination, which were selected for unbinding simulations.

Fig. S5. Complete unbinding for ATP and GTP from *Blastocystis* and pig SCS. The figure shows the AMBER GBSA energy difference (relative to the apo receptor) as a function of time from 0 to 5 ns for two different simulations (Free and Bound) for each receptor-ligand pair. In the Free simulation, the ligand was allowed to diffuse away from the receptor. In the Bound simulation, the ligand was restrained to its initial position by a harmonic penalty function, as described in Material and Methods. The top graph (A) shows the energies for the *Blastocystis* SCS model and the lower graph (B) shows the energies for the pig SCS receptor.

Fig. S6. Side-chain interactions with nucleotide. Stereo views of *Blastocystis* SCS bound to ATP (A) and GTP (B) and of pig SCS bound to GTP (C) are shown. Residues that make contact with the nucleotide group on the ligand are labeled. The motion of Gln 20, which stabilizes GTP in pig SCS, but moves out of the way to

accommodate the ATP amino group in the ATP-*Blastocystis* SCS model, can be seen by comparing figures A and B. The figures were prepared using Molscript v2.1.2 (Kraulis, 1991) and Raster3 (Merritt & Bacon, 1997).

References

Kraulis, P. J., (1991) Molscript - a Program to Produce Both Detailed and Schematic Plots of Protein Structures. *Journal of Applied Crystallography* **24**: 946-950.

Merritt, E. A. & D. J. Bacon, (1997) Raster3D: Photorealistic molecular graphics. *Macromolecular Crystallography, Pt B* **277**: 505-524.

Stechmann, A., K. Hamblin, V. Perez-Brocal, D. Gaston, G. S. Richmond, M. van der Giezen, C. G. Clark & A. J. Roger, (2008) Organelles in *Blastocystis* that blur the distinction between mitochondria and hydrogenosomes. *Curr Biol*: in press.

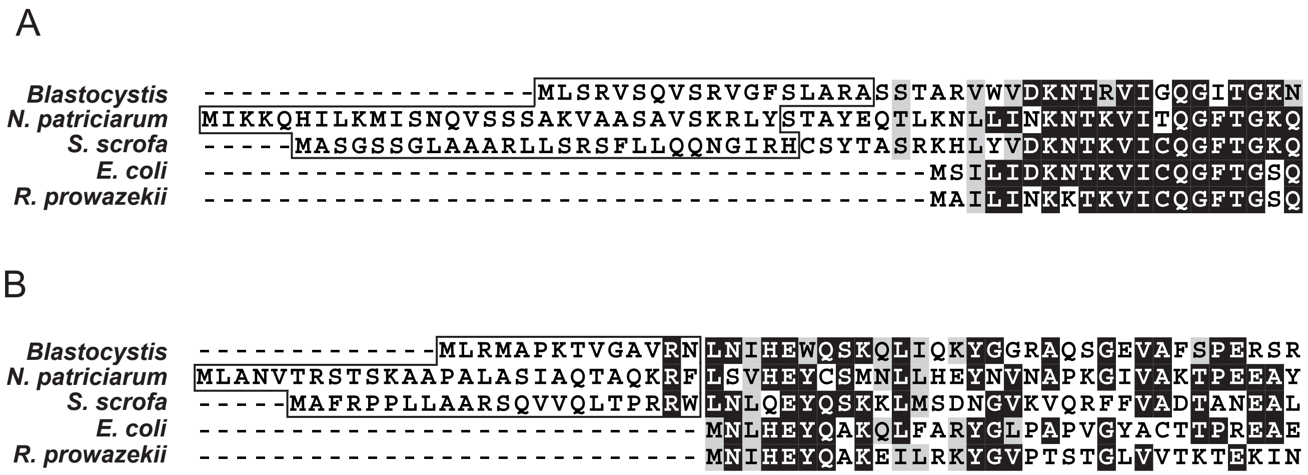
A
Blastocystis -----------------MLSRVSQVSRVGFSLARASSTARVWVDKNTRVIGQGITGKN
N. patriciarum MIKKQHILKMISNQVSSSAKVAASAVSKRLYSTAYEQTLKNLLINKNTKVITQGFTGKQ
S. scrofa -----MASGSSGLAAARLLSRSFLLQQNGIRHCSYTASRKHLYVDKNTKVICQGFTGKQ
E. coli -------------------------------------MSILIDKNTKVICQGFTGSQ
R. prowazekii -------------------------------------MAILINKKTKVICQGFTGSQ
B
Blastocystis -------------MLRMAPKTVGAVRNLNIHEWQSKQLIQKYGGRAQSGEVAFSPERSR
N. patriciarum MLANVTRSTSKAAPALASIAQTAQKRFLSVHEYCSMNLLHEYNVNAPKGIVAKTPEEAY
S. scrofa -----MAFRPPLLAARSQVVQLTPRRWLNLQEYQSKKLMSDNGVKVQRFFVADTANEAL
E. coli ---------------------------MNLHEYQAKQLFARYGLPAPVGYACTTPREAE
R. prowazekii ---------------------------MNIHEYQAKEILRKYGVPTSTGLVVTKTEKIN

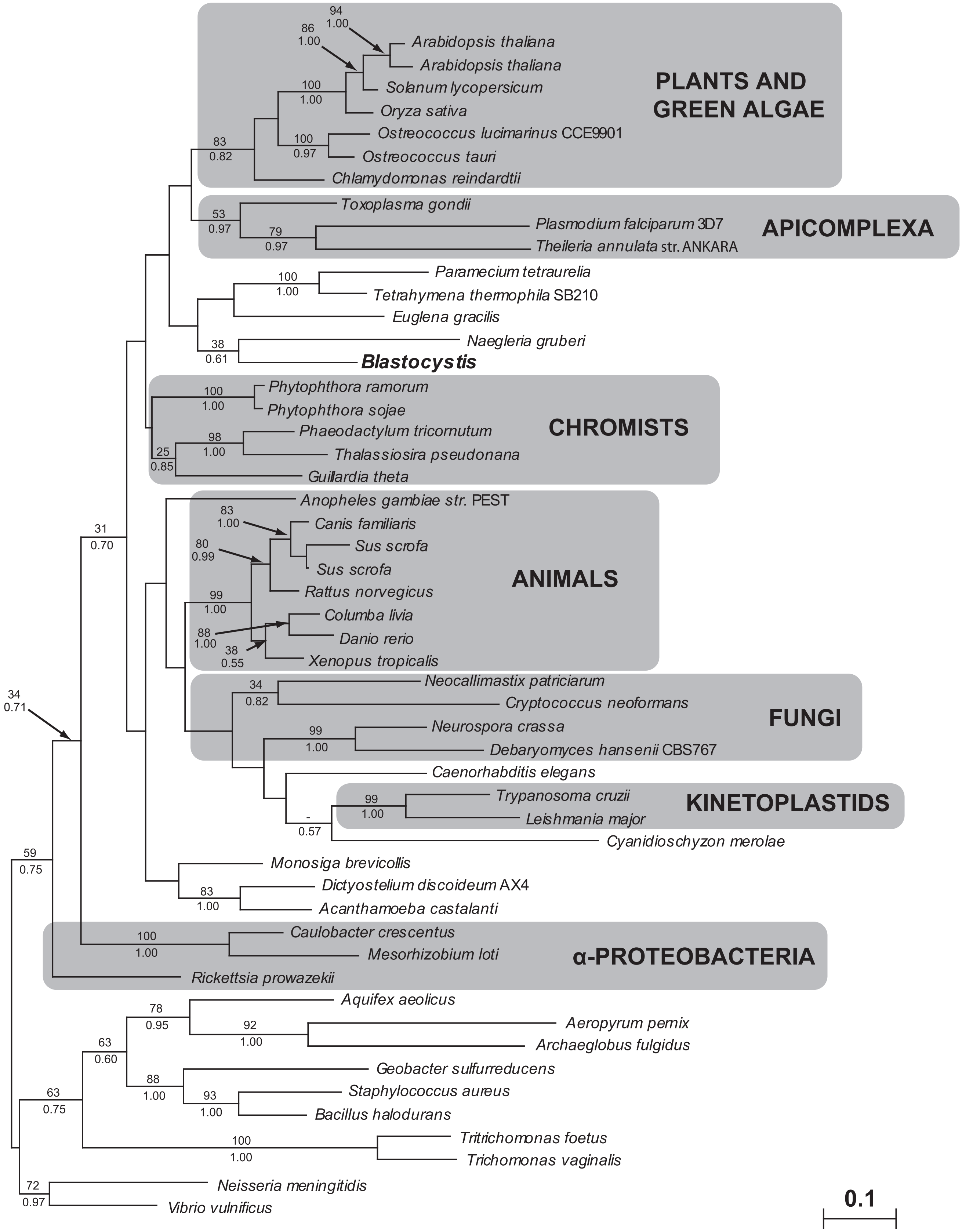

PLANTS AND GREEN ALGAE
Arabidopsis thaliana
Arabidopsis thaliana
Solanum lycopersicum
Oryza sativa
Ostreococcus lucimarinus CCE9901
Ostreococcus tauri
Chlamydomonas reindardtii
APICOMPLEXA
Toxoplasma gondii
Plasmodium falciparum 3D7
Theileria annulata str. ANKARA
Paramecium tetraurelia
Tetrahymena thermophila SB210
Euglena gracilis
Naegleria gruberi
Blastocystis
CHROMISTS
Phytophthora ramorum
Phytophthora sojae
Phaeodactylum tricornutum
Thalassiosira pseudonana
Guillardia theta
Anopheles gambiae str. PEST
ANIMALS
Canis familiaris
Sus scrofa
Sus scrofa
Rattus norvegicus
Columba livia
Danio rerio
Xenopus tropicalis
FUNGI
Neocallimastix patriciarum
Cryptococcus neoformans
Neurospora crassa
Debaryomyces hansenii CBS767
Caenorhabditis elegans
KINETOPLASTIDS
Trypanosoma cruzii
Leishmania major
Cyanidioschyzon merolae
Monosiga brevicollis
Dictyostelium discoideum AX4
Acanthamoeba castalanti
α-PROTEOBACTERIA
Caulobacter crescentus
Mesorhizobium loti
Rickettsia prowazekii
Aquifex aeolicus
Aeropyrum pernix
Archaeglobus fulgidus
Geobacter sulfurreducens
Staphylococcus aureus
Bacillus halodurans
Tritrichomonas foetus
Trichomonas vaginalis
Neisseria meningitidis
Vibrio vulnificus
0.1

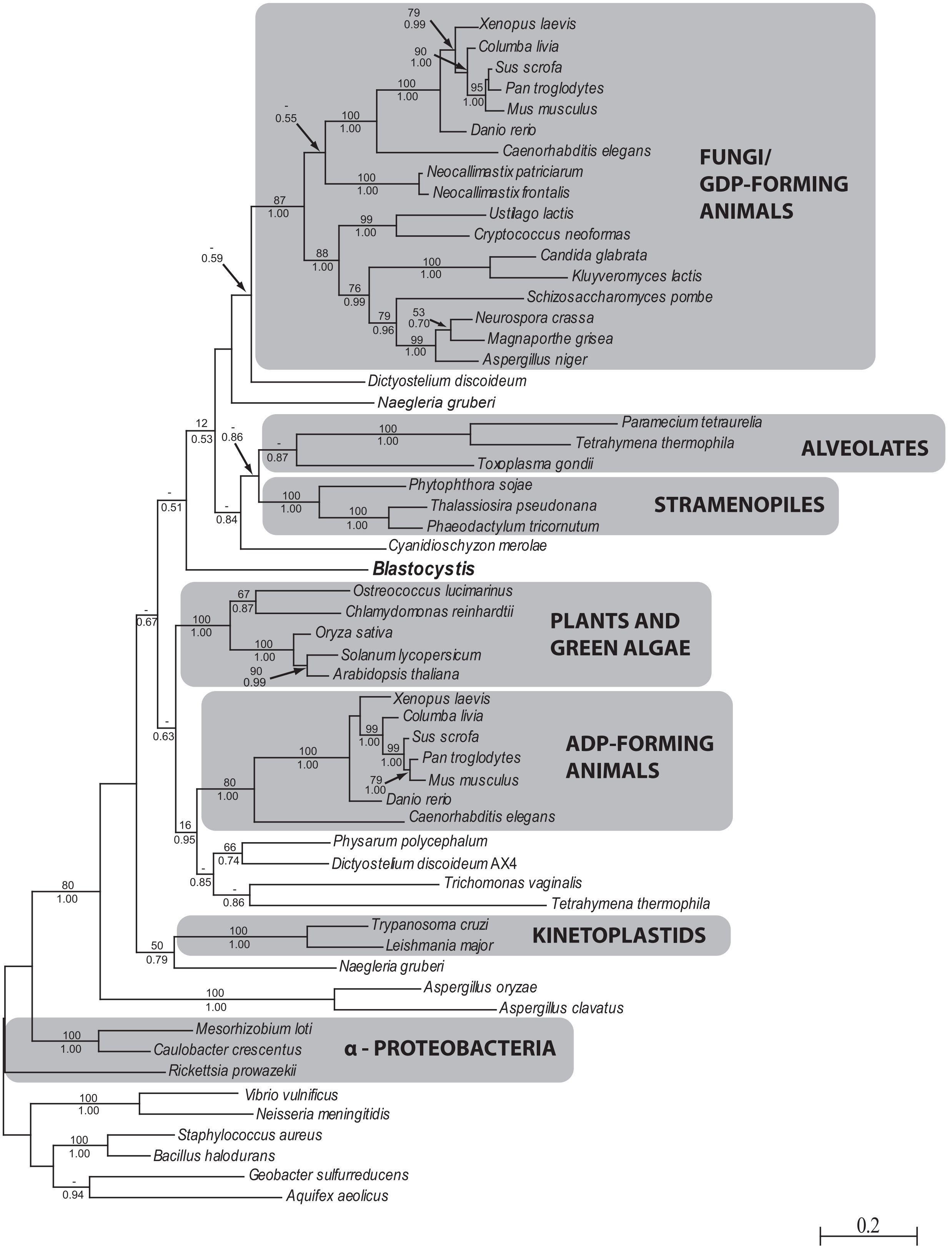

FUNGI/
GDP-FORMING
ANIMALS
Xenopus laevis
Columba livia
Sus scrofa
Pan troglodytes
Mus musculus
Danio rerio
Caenorhabditis elegans
Neocallimastix patriciarum
Neocallimastix frontalis
Ustilago lactis
Cryptococcus neoformas
Candida glabrata
Kluyveromyces lactis
Schizosaccharomyces pombe
Neurospora crassa
Magnaporthe grisea
Aspergillus niger
Dictyostelium discoideum
Naegleria gruberi
ALVEOLATES
Paramecium tetraurelia
Tetrahymena thermophila
Toxoplasma gondii
STRAMENOPILES
Phytophthora sojae
Thalassiosira pseudonana
Phaeodactylum tricornutum
Cyanidioschyzon merolae
Blastocystis
PLANTS AND
GREEN ALGAE
Ostreococcus lucimarinus
Chlamydomonas reinhardtii
Oryza sativa
Solanum lycopersicum
Arabidopsis thaliana
ADP-FORMING
ANIMALS
Xenopus laevis
Columba livia
Sus scrofa
Pan troglodytes
Mus musculus
Danio rerio
Caenorhabditis elegans
Physarum polycephalum
Dictyostelium discoideum AX4
Trichomonas vaginalis
Tetrahymena thermophila
KINETOPLASTIDS
Trypanosoma cruzi
Leishmania major
Naegleria gruberi
Aspergillus oryzae
Aspergillus clavatus
α - PROTEOBACTERIA
Mesorhizobium loti
Caulobacter crescentus
Rickettsia prowazekii
Vibrio vulnificus
Neisseria meningitidis
Staphylococcus aureus
Bacillus halodurans
Geobacter sulfurreducens
Aquifex aeolicus
0.2

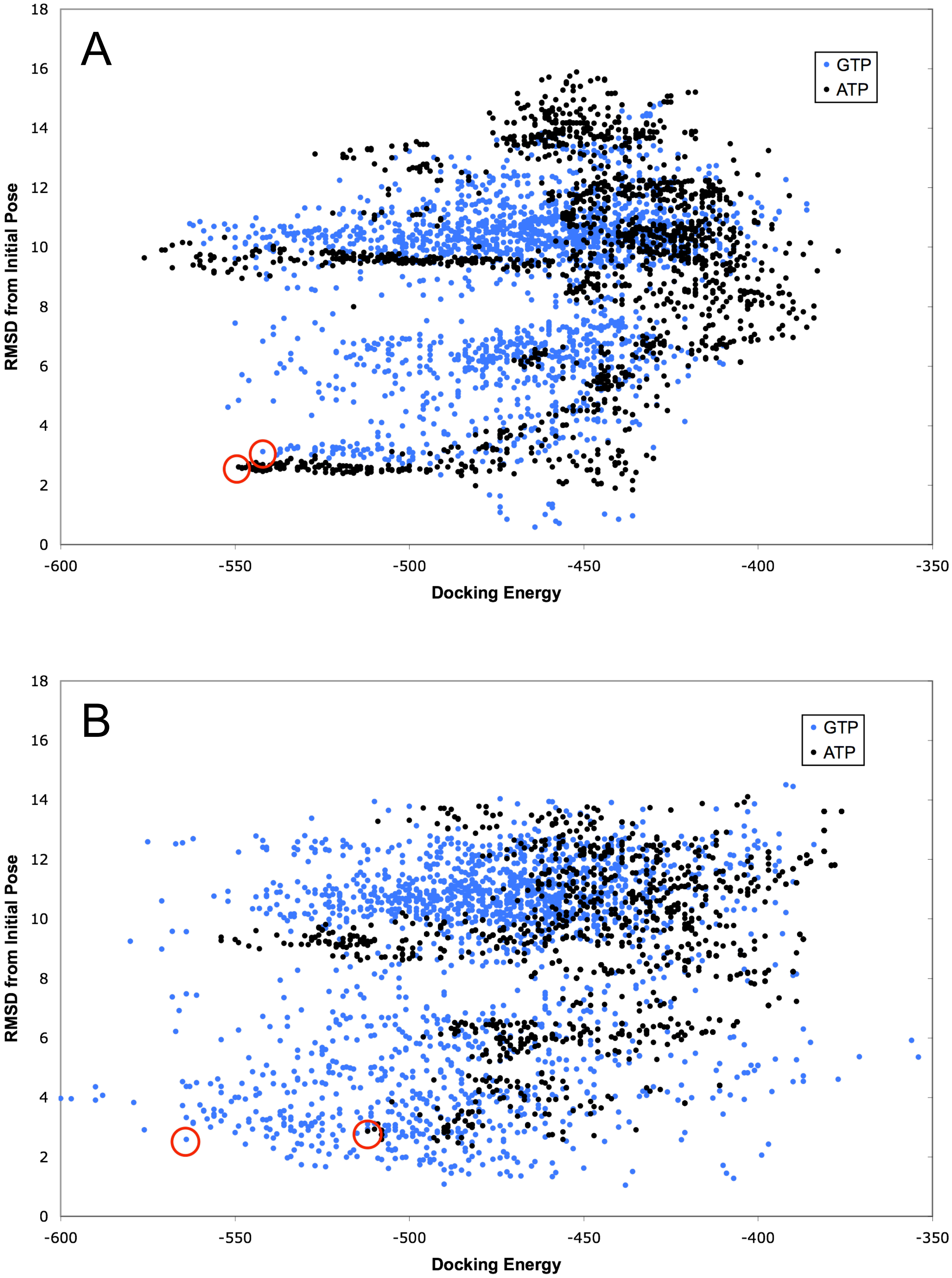
A
GTP
ATP
RMSD from Initial Pose
Docking Energy
0
2
4
6
8
10
12
14
16
18
-600
-550
-500
-450
-400
-350
B
GTP
ATP
RMSD from Initial Pose
Docking Energy
0
2
4
6
8
10
12
14
16
18
-600
-550
-500
-450
-400
-350

A

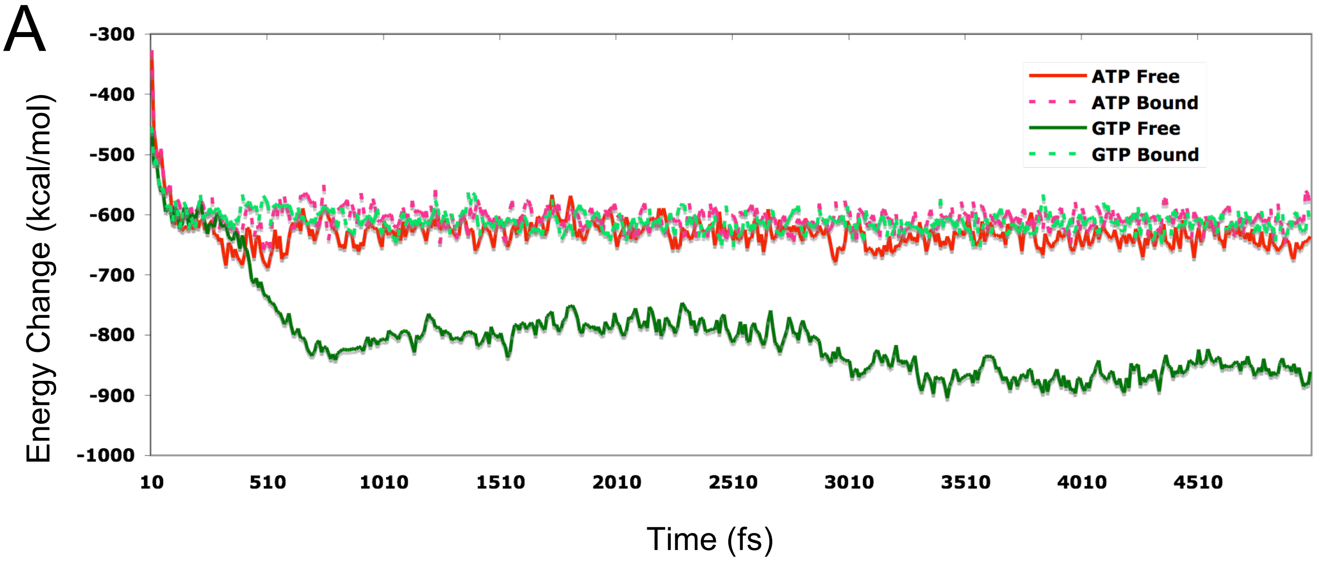

-300
-400
-500
-600
-700
-800
-900
-1000
Energy Change (kcal/mol)
10
510
1010
1510
2010
2510
3010
3510
4010
4510
Time (fs)
ATP Free
ATP Bound
GTP Free
GTP Bound

B

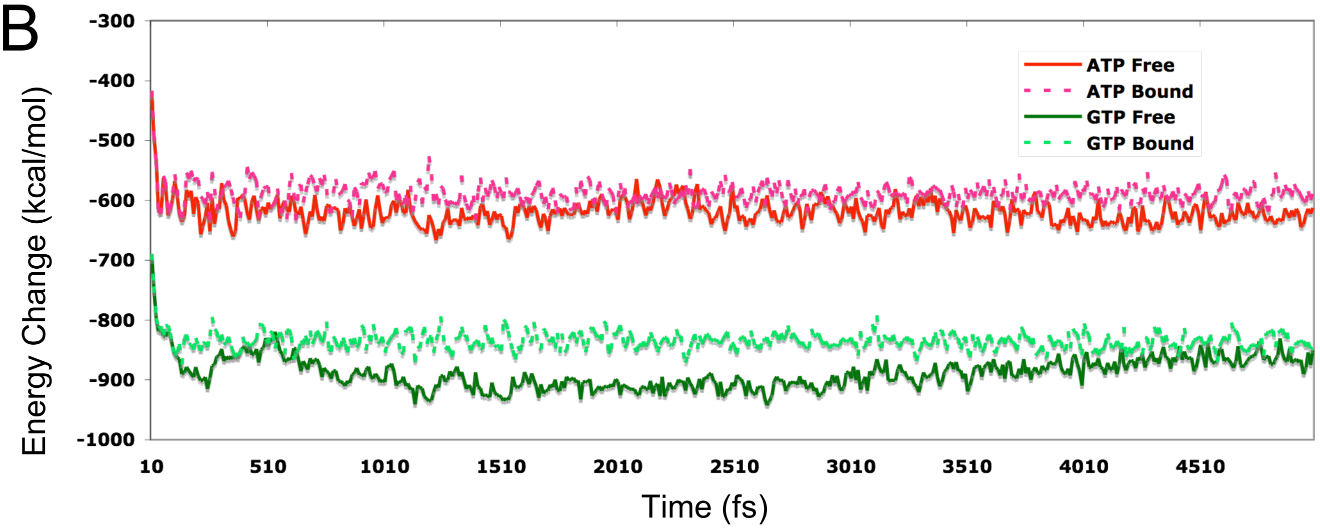

-300
-400
-500
-600
-700
-800
-900
-1000
Energy Change (kcal/mol)
10
510
1010
1510
2010
2510
3010
3510
4010
4510
Time (fs)
ATP Free
ATP Bound
GTP Free
GTP Bound

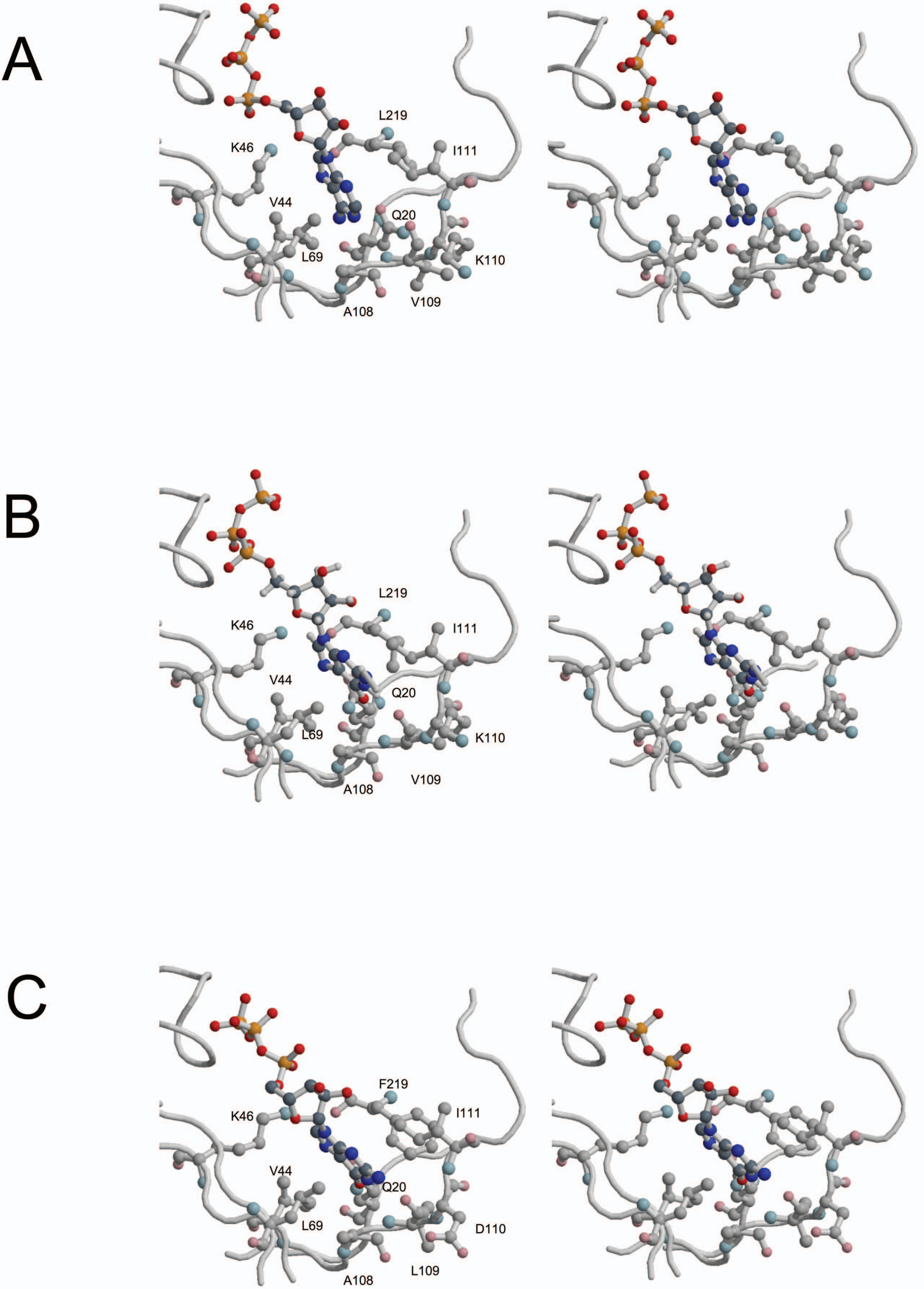
A
L219
K46
I111
V44
Q20
L69
K110
A108
V109
B
L219
K46
I111
V44
Q20
L69
K110
A108
V109
C
F219
K46
I111
V44
Q20
L69
D110
A108
L109
